# Mental health literacy and the public perception of persons with depression and schizophrenia in Vietnam

**DOI:** 10.3389/fpsyt.2024.1430272

**Published:** 2024-11-26

**Authors:** Mahan Mobashery, Thi Minh Tam Ta, Duc Tien Cao, Kerem Böge, Luisa Eilinghoff, Van Phi Nguyen, Selin Mavituna, Lukas Fuchs, Sebastian Weyn-Banningh, Solveig Kemna, Malek Bajbouj, Eric Hahn

**Affiliations:** ^1^ Department of Psychiatry and Neurosciences, Charité – Universitätsmedizin Berlin, Berlin, Germany; ^2^ Department of Psychiatry, Buon Ma Thuot Medical University, Buon Ma Thuot, Dak Lak, Vietnam; ^3^ Department of Psychiatry, Hanoi Medical University, Hanoi, Vietnam; ^4^ Department of Psychiatry, National Geriatric Hospital, Hanoi, Vietnam; ^5^ Department of Migration, German Centre for Integration and Migration Research (DeZIM), Berlin, Germany

**Keywords:** schizophrenia, depression, Vietnam, causal beliefs, stigma and awareness, mental health literacy, cross cultural psychiatry

## Abstract

**Background:**

Vietnam’s mental health care system is undergoing significant changes since the government has initiated large-scale programs to reform and develop the mental health care infrastructure. Cultural belief systems on mental illnesses influence help-seeking behavior and compliance. This study investigates the belief systems about people with schizophrenia and depression among people living in the Hanoi area.

**Method:**

1077 Vietnamese participants answered two open-ended questions after reading an unlabeled vignette describing a character with the symptoms of schizophrenia or depression. The answers were analyzed using thematic analysis.

**Results:**

Of all participants, 88,4% associated the presented cases with a mental illness, with 91,5% in the case of schizophrenia and 85,1% in the case of depression, so both disorders were conceptualized as mental illnesses. 18,6% mentioned depression when presented with the depression vignette, while only 3,6% recognized schizophrenia in the schizophrenia condition.

**Conclusions:**

Almost 9 out of 10 participants considered the presented cases as an example of mental illness, suggesting a high mental health awareness among our participants. The majority did not identify the presented cases as examples of schizophrenia or depression, reflecting little familiarity with Western mental health concepts. It could be interpreted as a sign of relatively low mental health literacy among the study participants.

## Introduction

The comprehensive mental health action plan of the World Health Organization (WHO) envisions the worldwide accessibility of high quality, culturally appropriate, adequate community-based mental health care and has developed a guideline to integrate mental health into primary health care (1). The WHO framework has influenced the assessment and progress of Vietnamese mental health care into primary care. Over the past two decades, the Vietnamese government has initiated large-scale programs to reform and develop the mental health care infrastructure (2, 3). The Ministry of Health (MoH) of Vietnam included mental health as one of the National Health Target Programs focusing on schizophrenia and epilepsy and announced a National Mental Health Strategy 2015–2025. Ongoing plans and measures to scale up mental health services and psychological interventions have been reinforced in the wake of the Covid-19 pandemic, as the Vietnamese government considers mental health care as an essential element of a resilient health system (decision No. 2057/QD-BYT of May 2020 on strengthening mental health care at COVID-19). The government plans to implement mental health care in a general strategy to improve preparedness for future global health risks (4).

However, services still primarily focus on psychopharmacological treatment, with psychotherapeutic treatment options being rare. There is a shortage of human resources in mental health (5). According to a WHO survey, there were 0.91 psychiatrists per 100,000 population in 2014 (6), an increase from 2006, with only 0.35 in 2006 (7). Historically, Vietnam’s healthcare system has shown a particularly pronounced focus on physical well-being, with mental healthcare only recently beginning to gain attention and integration. Psychiatric services and training in Vietnam are primarily hospital-based, and medical doctors’ psychiatric training includes diagnostics, psychopharmacology and psychosocial interventions but does not include structured psychotherapy training. Furthermore, the few psychologists working in the mental health system are primarily focused on neuropsychological assessments. Currently, only 143 clinical psychologists and psychotherapists work in public psychiatric hospitals across Vietnam (8).

In the context of a changing and developing mental health care system in Vietnam, knowledge of the population’s concepts on mental illness can inform and improve mental health awareness campaigns and help practitioners adapt how they communicate with patients. Concepts about mental illness vary within and across cultures and affect many aspects of psychiatric practice, such as help-seeking behavior, diagnosis, and perceived quality of care (9). Although there is a growing body of research on differences in the perception and interpretation of mental illnesses, there is still a critical knowledge gap concerning variations in experiences of mental illnesses in non-European cultures. As Fernando (10) highlights *“what stands out is the lack of any attention to what users of services may think or to what communities, where Western psychiatric models of health are alien concepts, may wish to see for improving their mental health and wellbeing”* (p.22). This study aims to contribute to a growing body of research on non-Western perspectives on mental health by analyzing the perception of two disorders, schizophrenia and depression, in a Vietnamese sample from the Hanoi region of Vietnam. The Hanoi region encompasses both, rural and urban areas, and more than 50% of our participants lived in rural areas, which allowed for a geographically diverse sample, encompassing both urban and rural participants. In the context of Vietnamese patients, investigating the perception of and attitudes towards mental illness is essential to inform the ongoing development and transformation of the mental health care system. It is also of interest because mental health service utilization appears low among Vietnamese communities, especially among first-generation migrants in Western countries such as Australia, Canada, Germany, Norway and the USA (11–14). Several authors have noted that there is a strong stigma towards mental illness among Vietnamese migrants (15, 16) and that they and their families are often disgraced, which makes them reluctant to seek help for mental health issues (17).

### Purpose

Drawing on research on perceived causes of schizophrenia and depression and their relationship to social distance (18–20), this study aims to explore attitudes of Vietnamese participants of the Hanoi region towards people with the symptoms of these two disorders. While most studies explored perceived causes of mental illnesses by introducing a list of possible explanations, we asked open-ended questions that allowed respondents to answer in an open-text format. Open-ended questions can provide valuable information on the respondents’ point of view without limiting the answers to fixed alternatives and elicit the respondent’s beliefs without *a priori* assumptions (21, 22). By presenting cases of schizophrenia and depression, we aim to assess whether the described circumstances are considered illnesses, if they are described in terms equivalent to a Western understanding of mental health care, and to investigate possible differences in attitudes towards the two disorders.

## Methods

### Sample description

Data collection was conducted between April and August 2013 in Hanoi. Hanoi has over 7,000,000 inhabitants, living in 11 urban and 18 rural districts (23). Participants were recruited through the medical students and the staff at the Psychiatric Department of Hospital 103, Military Academy of Medicine. While the questionnaires were distributed by staff from Hospital 103, these participants were not directly contacted by members of the Department of Psychiatry or the study team. Questionnaires were disseminated through wider networks of social contacts. There was no direct contact between the research team and the participants. Additionally, only one person per household was allowed to complete the questionnaire.

The study was approved by the ethics committee of the Department of Psychiatry and Psychological Medicine, Hospital 103, Military Academy of Medicine, Hanoi, Vietnam. Data collection involved only healthy participants in Vietnam in this population-based health survey, and participation was entirely voluntary. All participants remained fully anonymous to the research team, with personal data limited to the sociodemographic details of the study sample as provided in the results and no personal benefit. It was assessed that no potential harm was associated with participating in the study. All participants provided written informed consent for their voluntary participation.

Many of these recruited people had access to potential multiplicators to distribute the questionnaires further, and a quota sampling approach was used. There was no financial compensation. All subjects received written information about the research and its purposes and had to give their signed consent. In total, more than 1200 questionnaires were distributed equally attached with either a vignette illustrating symptoms of schizophrenia or depression in a man or woman. The returned questionnaires were then examined for errors and missing data, and 1077 participants were entered into the analysis.

### Procedure

#### Vignettes

The participants received vignettes depicting a person with ICD-10 symptoms of either schizophrenia or depression and were distributed in a version either with a male or a female protagonist. The same vignettes have been used and reaffirmed in several previous studies (24, 25). Participants were asked the following questions and were asked to answer freely: *How would you describe the condition of this woman/man? What do you think this woman/man is suffering from?*

#### Translation procedure

A native Vietnamese speaker and certified psychiatrist translated and adapted the vignettes and the questionnaire from German into Vietnamese. An independent bilingual German-Vietnamese psychologist then back-translated the translation into German. Both versions were discussed and revised with the assistance of an independent translator proficient in both Vietnamese and German who did not work in the mental health field. This translator was hired by the research team from the Department of Psychiatry at Military Hospital 103 in Hanoi. The group discussion between these translators also involved psychiatrists from the research team at the Department of Psychiatry, Military Hospital 103, who served as field specialists. Their role was to ensure that the meaning of the questionnaire was preserved and that the final translation was linguistically and contextually appropriate for Vietnamese individuals living in the Red River Delta catchment area. The questionnaire and the vignettes have been used successfully in previous studies (26–28).

All replies were translated into English by two psychologists who speak both English and Vietnamese for the writing of this paper and were discussed with the last author of the article in case of ambiguity.

#### Coding of categories

We used the thematic analysis method (29) and followed the suggested six phases of analysis to categorize the answers. The first author of this study familiarized himself with the entire data by reading the entire data set. We chose a semantic approach as there is little research on public opinions towards mental health in Vietnam. Any assumptions going beyond what participants answered using other sources of information, such as previous research, would be difficult to justify. In line with this, the coding scheme was data-driven, not theory-driven. Every concept mentioned was coded into one category using an inductive approach (30), resulting in 160 categories in the qualitative research software MaxQDA Version 20. For example, we counted the number of participants who mentioned that the mentally ill protagonist described in the vignette would benefit from the support of relatives (one category) or the support of a friend (another category). In the next step, these initial categories would then be merged into themes; in the example above, it would be “support of close people”, or to give another example, “treatment by a psychiatrist or psychologist” would be summarized under “treatment by a mental health practitioner”. These larger categories were associated with four main themes: Diagnoses, description of the vignette’s protagonist, perceived causes, and treatment recommendations. The themes were reviewed and discussed with the co-authors to ensure they did not overlap and were subsequently named.

In a second step we analyzed frequencies of themes. Braun and Clarke (31), mention that frequency counts can complement thematic analysis. The validity of counting in qualitative research is controversial and depends on numerous factors (32). However, several aspects of our study ensure that counting does produce relevant information. First, the sample size is large enough to distinguish between response patterns mentioned by almost all participants. Second, to have a large sample to maintain comparability between the answers, our questions were standardized, there were no follow-up questions asked. Thus, fitting a larger sample size, this study design ensured that interviewers did not further influence the content participants’ answers. Finally, since participants all read a vignette and were only asked two questions, our participants’ responses were concise. It did not require much interpretation by the coders, for example, whether or not a participant mentioned a treatment like psychotherapy as a possibly helpful intervention. We made sure that we maintained a distinction between the importance of the codes from their frequency of common or related occurrence in order to not omit relevant themes, only because they have been mentioned rarely (33).

A second coder rated 5 percent of the entire data. Cohens Kappa was calculated as a measure of coder agreement (kappa=0.9). The high overall coder agreement is partially due to the participants’ straightforward answers; thus, there was not much ambiguity and difficulties of interpretation.

#### Statistical analysis

Cohen’s Kappa was calculated as a measure of coder agreement. Chi-square tests were applied to investigate if and how either the diagnosis (schizophrenia, depression), the gender of the participant, or the gender of the vignette’s protagonist influenced the answers (the “choice” of a category). The Fisher Exact test was executed when the expected frequency in one cell was lower than five.

## Results

### Sociodemographics

All sociodemographic data concerning the study sample are presented in **Table 1** **next to results of the** *Hanoi Census (2009) and the more recent micro-census (2013)*, corresponding to the sampling timeframe.

**Table 1:** Sociodemographic characteristics of the participants.

Sample characteristics	Total survey sample %	Population of Hanoi (n=6,936,900) %
Gender^a^		
Male	52,4	49.8
Female	47,5	50.2
Urbanity^a^		
Urban	45,5	42.5
Rural	54,5	57.5
Age years^b^		
16-19	15.3	14.9
20-24	30.5	14
25-29	17.8	13
30-34	9.5	11.4
35-39	7.7	11.1
40-44	6.7	10.2
45-49	4.9	9.2
50-54	5.2	7.7
55-59	1.3	5.2
60-64	1.0	3.3
Household size^b^		
1	8,4	9.1
2-4	58.9	70.2
5-6	25.1	18.7
7+	7,7	2.0
Unemployment rate^c^		
Total	3.8	2.5

N=1077 All data are given in percentage. a Data from micro-census 2013 (23). b Data from complete census 2009 (23). c Education for All 2015 Review Report: Viet Nam (UNESCO & Ministry of Education and Training Vietnam, 2015).

### Categories

When analyzing the answers, we obtained 95 categories. Although we had only asked the participants what the character in the vignette was suffering from, we still received many responses that referred to perceived causes and possible treatments. We could organize the answers into the following meta-categories: 1. Diagnoses (**Table 2**), 2. Description of the vignette’s protagonist (**Table 3**), 3. Perceived causes (**Table 4**), 4. Treatment recommendations (**Table 5**).

**Table 2:** Frequencies of diagnoses.

Diagnosis	Schizophrenia Vignette (n=552)		Depression Vignette (n=525)		X
	%	n	%	n	
Psychological illness, psychological problem (bệnh tâm lý)	15,2	84	16,2	85	*non-significant*
Psychological disorder (rối loạn tâm lý)	5,4	30	4,4	23	*non-significant*
Psychiatric problem/Illness (Bệnh tâm thần)	25,7	142	13,9	73	*χ* ^2^, 1, *n* = 1077, 23,52 *p* <.001).
Neurological problem/Illness (bệnh thần kinh)	24.1	133	17	89	*χ* ^2^, 1, *n* = 1077, 8,39 *p* = .004).
Depression (trầm cảm)	8,7	48	18,7	98	*χ* ^2^, 1, *n* = 1077, 23,67 *p* <.001)
Schizophrenia (bệnh tâm thần phân liệt)	3,6	20	0,6	3	*χ* ^2^, 1, *n* = 1077, 11,99 *p* <.001
Delusion (hoang tưởng)	14,5	80	1,9	10	*χ* ^2^, 1, *n* = 1077, 55,67 *p* <.001
No reference to mental illness	8,5	47	14,9	78	*χ* ^2^, 1, *n* = 1077, 10,55 *p* <.001).
Autism (tự kỷ.)	2,4	13	1,5	8	*non-significant*

% of all participants in the schizophrenia (n=552) or depression condition (n=525) (the most frequent Vietnamese word or term used by the participants is indicated in brackets).

**Table 3:** Frequencies of different descriptions of the vignette’s protagonist.

Description	Schizophrenia Vignette (n=552)		Depression Vignette (n=525)		X
	%	n	%		
not normal (không bình thường)	15,2	84	12,4	65	*non-significant*
not stable (không ổn định)	4,3	24	5,9	31	*non-significant*
Sad (buồn rầu)	1,4	8	4,2	22	*χ* ^2^, 1, *n* = 1077, 7,46 *p*=.006).
Hopeless (tuyệt vọng)	0,2	1	3,2	17	*χ* ^2^, 1, *n* = 1077, 15,30 *p* <.001). Fisher Exact Test
Has wrong/unfound thoughts (suy nghĩ không đúng/suy nghĩ không minh mẫn)	3,6	20	1,1	6	*χ* ^2^, 1, *n* = 1077, 7,03 *p*=.008).
Has not an illness/ it’s normal (bình thường)	2,4	13	3	16	*non-significant*
Mad (phát điên/điên loạn)	0,5	3	0	0	*non-significant*

% of all participants in the schizophrenia (n=552) or depression condition (n=525) (the most frequent Vietnamese word or term used by the participants is indicated in brackets).

**Table 4:** Frequency of perceived causes.

Cause	Schizophrenia Vignette (n=552)		Depression Vignette (n=525)		X
	%	n	%	n	
Stress (Stress/căng thẳng về thần kinh)	9,6	53	19,6	103	χ2, 1, n = 1077, 21,800 p.000
Work Issues	8,2	45	16,2	85	χ2, 1, n = 1077, 16,31 p <.000
Worries	3,1	17	9	47	χ2, 1, n = 1077, 4,475 p= .034)
Tired/Lack of sleep	0,7	4	5,9	31	χ2, 1, n = 1077, 18,79 p <.001
Family Problems	2,9	16	5,1	27	*non-significant*
All difficult life events	7,2	40	10,1	53	*non-significant*
Thinking too much	1,8	10	5,1	27	χ2, 1, n = 1077, 9,00 p=.003
Trauma/Shock (sang chấn tâm lý/ tâm lý quá lớn)	2,1	12	1.1	6	*non-significant*
Not believe in oneself/ Inferiority Complex	3,4	19	4,4	23	*non-significant*
Wrongdoings and critical remarks on the vignette ‘s protagonist	4,5	25	1	5	*χ* ^2^, 1, *n* = 1077, 12,71 *p*<.001).
Possessed	0,9	5	0	0	*non-significant*

% of all participants in the schizophrenia (n=552) or depression condition (n=525) (the most frequent Vietnamese word or term used by the participants is indicated in brackets).

**Table 5:** Frequency of treatment recommendations.

Recommendation	Schizophrenia Vignette (n=552)		Depression Vignette (N=525)		X
	%	n	%	n	
Any kind of medical or psychological treatment	18,7	103	17,1	90	*non-significant*
Medical treatment	15,2	84	11,05	58	*χ* ^2^, 1, *n* = 1077, 4,08 *p* = .043
See a psychologist (chuyên gia tâm lý)	3,4	19	6,1	32	*χ* ^2^, 1, *n* = 1077, 4,2 *p* = .040
See a specialist/doctor	16,3	90	4,9	26	*χ* ^2^, 1, *n* = 1077, 36,08 *p* <.001
See a Psychiatrist (Cần đi khám bác sĩ tâm thần)	1,3	7	0,6	3	*non-significant*
Hospital Examination/ Medical facility (cần được đi khám ở cơ sở y tế)	2,9	16	3,6	19	*non-significant*
Support of surrounding people	3,8	21	2,9	15	*non-significant*
Needs rest	0,5	3	1,5	8	*non-significant*

% of all participants in the schizophrenia (n=552) or depression condition (n=525) (the most frequent Vietnamese word or term used by the participants is indicated in brackets).

#### Diagnoses

The term “psychological illness” *(bệnh tâm lý)* was used more than twice as often as “psychological disorder” *(rối loạn tâm lý)*. Still, there was no significant difference in using these terms regarding a correlation with gender or the diagnosis. In the schizophrenia condition, 24% of the participants explicitly mentioned a psychological or mental illness. In the depression condition, it was 25%. Twice as many participants mentioned the term for a psychiatric disorder in the schizophrenia condition. Almost twice as many participants diagnosed the patient with schizophrenia with a neurological or brain-associated or neurological *(thần kinh)* problem. On the other hand, almost twice as many participants mentioned depression (trầm cảm) when the depression vignette was presented (18,7%) compared to the schizophrenia vignette (8,7%). Only three participants (0,6%) diagnosed the depressed protagonist as schizophrenic *(tâm thần phân liệt)* compared to 20 (3,6%) in the schizophrenia condition that diagnosed the person in the vignette as depressed. Another psychiatric term was used far more frequently for the schizophrenic character, *hoang tưởng*, which could be translated as paranoia or delusion. 14,5% used it for the person with schizophrenia and only 1,9% for the person depicted in the depression vignette.

*“This person is in fear and paranoia, scaring himself.” (Người này đang trong trạng thái lo sợ và hoang tưởng. Suy nghĩ tự làm mình sợ.) (Answer in the schizophrenia condition).*

Autism (tự kỉ) was mentioned in both conditions, 13 times for schizophrenia and 8 times for depression. Only a minority in both conditions did not refer to mental illness in any way (8,5% for schizophrenia and 14,9% for depression). Typical answers among these refer to difficult life circumstances. Thus, 88,4% of the participants associated the presented cases with a mental illness, 91% in the case of schizophrenia, and 85,1% in the case of depression.

“This is common for people in Vietnam in general due to the difficult economic situation; people must *think more about work to ensure life for their families.” (đây là sự thể thường gặp ở người Việt Nam nói chung do nền kinh tế khó khăn, mọi người phải suy nghĩ nhiều hơn cho công việc để đảm bảo cuộc sống cho gia đình mình.”)*

*(Answer in the depression condition).*

#### Descriptions of the vignette’s protagonist

The description “not normal*” (không bình thường)* was mentioned often in both conditions. Another broadly used non-medical term to describe the vignette protagonist was that the person is perceived as “not in a stable condition” (*không ổn định).* The opposite answer that the depicted person was perceived as “normal” (bình thường) was rare. In the depression condition, a larger number described the character as hopeless, sad, or worried. More participants considered the person in the schizophrenia vignette to display “not right thinking” *(suy nghĩ không đúng).* Only a few participants endorsed that the person was “normal” or that there were no signs of an illness.

#### Perceived *causes*

Although we did not ask for potential causes of the vignette’s character’s state, many participants gave possible explanations. “Stress”, “nervous tension”, or “pressure of life” were mentioned most often and about twice as often for depression. The English term “stress” was used 18 times, but most participants used terms like “psychological pressure” *(áp lực tâm lý nào đó)*, “nervous tension” *(căng thẳng về thần kinh)* or “pressure of life” *(vì áp lực cuộc sống).*

“Work issues” were mentioned in both vignettes. In the depression condition, the vignette only briefly referenced “concentration issues at work”. Nonetheless, more participants mentioned “problems at work” as a cause for the state of the vignette’s protagonist in the depression condition. Equally, “worries” and “being tired” were seen as causes more often in the depression condition.

“Family issues” or often “family pressure” were mentioned as a potential cause in both conditions*. “There is a family problem which she cannot solve” (có trục trặc về gia đình không giải quyết được)*. When “family pressure” was mentioned, it was usually mentioned along with “work stress”.

*“It could be due to family or professional pressure”*. *(Có thể là do áp lực trong gia đình hoặc nghề nghiệp).*

“Thinking too much” *(do suy nghĩ quá nhiều)* was mentioned significantly more often in the depression condition. As an example, one participant in the schizophrenia condition said:

*“According to me, this person is thinking a lot, and this leads to neurological disorders”*. *(Theo tôi người này suy nghĩ nhiều và dẫn đến rối loạn thần kinh)*.

While the vignette mentioned sleeping problems as a symptom, “lack of sleep” was often mentioned as a cause for the depressed character.

Most participants who referred to difficult life events did not specify the type of event. Trauma and shock were mentioned more often in the schizophrenia condition. Only a few participants (n=30) were critical of the vignette’s protagonist in one way or another. In the schizophrenia condition, the critical remarks mainly suggested that the person might have done something wrong (n=12).

*“do something bad/maybe this person did something wrong”*. *(làm một điều không tốt/thể người này đã làm việc gì đó sai phạm luôn)*.

and as a consequence, felt guilt (2%, n=9). The examples given were “committing a crime” (n=2) and “cheating on the partner” (n=2). Four participants said the protagonist was dangerous. Other answers included that the illness was due to watching too many movies, using drugs, superstition or that the person was overdramatizing (n=1 each). The critical remarks concerning the depressed character were, “This person has an ideological problem” (n=1), “lacks intellectual capacities” (n=1), “is decadent” (n=1), “creates his problem himself” (n=2), “harms people around” (n=1).

#### Treatment recommendations

In both conditions, any medical or psychological treatment was explicitly recommended overall in 18% of the cases. 13% of the respondents mentioned any medical treatment except psychotherapy, significantly more often in the schizophrenia condition. On the other hand, psychotherapy was recommended more often in the depression condition. Still, psychotherapy was mentioned rarely, and the difference was not significant. Only two participants claimed a need for medication for schizophrenia and four in depression (not shown in the table). In both conditions, a small percentage recommended support surrounding people, either friends or family. Other answers were that the vignette’s protagonist should do more activities, travel, or eat (n=1 each).

#### Gender *effects*

As shown in **Table 6**, both the gender of the vignette’s protagonist and the participants’ gender had an effect on claiming the described person was “not normal”. The male gender of the vignette’s protagonist was more likely to evoke that description. Additionally, male participants were slightly more likely to use it. When analyzed separately for the depression and schizophrenia vignettes, the effect of male participants in the schizophrenia condition on claiming the vignette’s character was “not normal” was significant. Female participants were more likely to recommend a psychologist in the depression condition, while the female-gendered vignettes were more likely to elicit this response. The effect was present in both conditions individually but only significant when tested over both conditions combined. Also, female participants were more likely to see the presented case as an example of a “psychological problem”.

**Table 6:** Chi square table for gender effects.

Category	Male Vignette % (n)	Female Vignette % (n)	X	Male participants % (n)	Female Participants % (n)	X
Not normal^1^	8,5%(92)	5,39% (58)	*χ* ^2^, 1, *n* = 1077, 10,564 *p* = .001).	8,64%(93)	5,29%(57)	*χ* ^2^, 1, *n* = 1077, 6,59 *p* = .037).
Not normal (schizophrenia condition)^2^	10,33% (57)	4,9% (27)	n = 552, *non-significant*	9,96% (55)	5,25% (29)	*χ* ^2^, 1, *n* = 552, 6, 84 p=.009
Should see a psychologist^1^	1,8%(19)	3,16%(34)	χ2, 1, n = 1077, 4,54 p= .033).	1,58%(17)	3,25%(35)	*χ* ^2^, 1, *n* = 1077, 9,56 *p= .*014).
Should see a psychologist (depression condition) ^3^	1,9%(10)	2,29% (12)	*n* = 525, *non-significant*	1,3%(7)	4,76% (25)	*χ* ^2^, 1, *n* = 525, 6,9 p=0,32
Should go to a hospital or medical center	2,4% (22)	1,21% (13)	n=1077 *non-significant*	2,4% (22)	1,21% (13)	n=1077 *non-significant*
Neurological, psychobiological^1^	8,54%(92)	5,85%(63)	*χ* ^2^, 1, *n* = 1077, 7,731, *p= .*005).	8,54%(92)	5,85%(63)	*non-significant*
Neurological, psychobiological (depression condition) ^3^	4,38% (23)	2,67%(14)	*χ* ^2^, 1, n = 525, 4,443 p=.035	4,57%(24)	2,48%(13)	χ2, 1, n = 525, 7,06 p=.029
Psychological Problem^1^	21,8%(115)	26,2%	ns.	20,3%(114)	28,3%(145)	*χ* ^2^, 1, *n* = 1077, 9,98 *p= .*007).
Psychological Problem (depression condition ) ^3^	8,95%(47)	15,81%(83)	*χ* ^2^, 1, n = 525, 6,117 p=.013	8,76%(46)	16%(84)	χ2, 1, n = 525, 5,65 p=.059 *non-significant*
Psychological Problem (schizophrenia) ^2^	12,31%(68)	11,05%(61)	*non-significant*	12,31%(68)	11,05%(61)	χ2, 1, n = 552, 4,076 p=.043
Neurological, Psychological, Psychiatric Problem combined (schizophrenia condition) ^2^	38,27%(210)	28,44%(157)	χ2, 1, n = 552, 10,393 p= .001).	38,22%(211)	28,26%(156)	*non-significant*
Wrong Thoughts^1^	1,7%(19)	0,84%(9)	*χ* ^2^, 1, *n* = 1077, 4,09 *p= .*043).	1,4%(15)	1,21(13)	*non-significant*
Wrong Thoughts (depression condition) ^3^	1,52%(8)	0%(0)	*χ* ^2^, 1, n = 525, 9,721 p=.002	1,14% (6)	0,38%(2)	*non-significant*
Possessed	(4)	1	*non-significant*	5	0	*non-significant*

1 Percent of all participants 2 Percent of participants in the schizophrenia condition 3 percent of participants in the depression condition.

Moreover, the female vignette elicited this response more often in the depression condition. Male participants framed it more often as a “neurological problem”. The latter effect was significant only in the depression condition, but the results in the schizophrenia condition pointed in the same direction. The male schizophrenia vignette was more likely to evoke any medical or psychological term, which appears to be in line with the fact that the symptoms described in the female vignette were more likely to be considered “normal”. This finding corresponds with the fact that only the male vignette in the depression condition led to the answer that the character had “wrong thoughts”.

## Discussion

### Mental health literacy in Vietnam

A high percentage of the participants recognized the presented cases as examples of mental illnesses. References to some mental illness were made from 91,5% of participants in the case of schizophrenia and 85,1% in the case of depression. This number certainly is high because participants knew Vietnamese mental health professionals led the study. Still, the difference between the conditions reflects variation in the perception of the different symptom patterns. In a study by Martensen (27) using the same vignettes in Vietnam but a questionnaire instead of open-ended questions, 85% of respondents endorsed that the person depicted in the schizophrenia vignette had a mental illness, while only 60% assumed the same in the case of depression. The fact that in this study, any reference to mental illness was counted, while Martensen (27) reports only the positive answers to the item “suffering from a mental illness in a medical sense” might account for that difference.

However, only a few ascribed the “correct” DSM diagnosis to the vignettes.

Jorm et al. (34) have defined mental health literacy “as the ability to recognize specific disorders, knowledge of risk factors and causes(…) and attitudes that promote recognition and appropriate help-seeking”. The concept is often operationalized as correctly identifying mental disorders in a presented vignette (34, 35). This means mental health literacy is defined as familiarity with Western concepts of mental illnesses. In this sense, there was a high perceived need for psychiatric or psychological care among the participants of the Hanoi region in our study, which also implies the perception of the presented character as “being ill”. However, it cannot solely be interpreted as comprehensive mental health literacy, since twice as many participants in the schizophrenia condition stated the person in the vignette was having depression rather than schizophrenia. The term depression seems to be thus used by some without knowledge about the symptoms it refers to. The term schizophrenia itself appears to be less familiar to our participants. The term was used only by 2,1% of all participants in both conditions and only 3,6% of all participants in the schizophrenia condition. Nevertheless, most participants interpreted the presented case as a mental illness. 46 participants (8,3%) did not mention any concept referring to mental illness at all.

Awareness and knowledge of mental illness regarding Western psychiatric concepts have likely increased over the past decade with the large-scale programs to develop the mental healthcare structure of Vietnam quoted in the introduction. The results in our study appear to reflect increasing mental health awareness when we compare it to results from other studies with Vietnamese participants (36–38). The emphasis on addressing depression and autism disorders in some of the programs aimed at improving mental health awareness in Vietnam may have influenced the high frequency of mentions of these disorders compared to schizophrenia in our study. In a study by Dang et al. (39), Vietnamese mental health professionals perceived misrepresentation of mental health-related subjects in Vietnamese media, particularly an over-representation of autism. In a qualitative part of a study by UNICEF (40), all participants stated that psychosocial and mental health problems were widespread and increasing, which could reflect growing knowledge of Western mental health care practice rather than an actual increase in the frequency of mental illnesses. Mental health literacy will most likely rise by increasing public awareness through educational campaigns, including mental health education in school curricula and training of healthcare professionals.

### Diagnoses, descriptions of the vignette’s protagonist and causal beliefs

There was a higher tendency to describe the schizophrenia case as a “mental illness”, a “psychiatric problem”, or “neurological problem”. This may be because the described symptoms are less frequent than those of depression and thus present a stronger deviation from what is considered “normal” (“not normal” was mentioned more often in the schizophrenia condition).

Many categories in both conditions align with the main categories found in other semi-qualitative or qualitative studies on mental illness in Vietnam (41, 42). “Stressful life” or “pressure of life” were the causes mentioned most often when the answers that could be considered as a diagnosis (like “psychiatric illness” or “depression”) were not counted. These findings are in line with Kamimura et al. (43). “Stress of life” was the cause mentioned most often by the Vietnamese participants (77.1%), while the participants in the US chose “chemical imbalance” (42%) most frequently. Autism was mentioned a few times in both conditions with no significant difference in frequency, suggesting that autism may be perceived as a mental illness without a clear distinction as to which symptoms would define it.

In contrast to some other studies, where a large part of the Vietnamese participants referred to concepts such as “bad karma”, “wrongdoing in the past”, or “being possessed by an evil spirit” when presented with cases of mental illnesses (43–46), those descriptions occurred infrequently in this study. Even though many authors suggest that supernatural, religious, and magical approaches to mental illness are common in Vietnam (43, 47), our findings did not point in this direction. A reason for the relatively low amount of answers reflecting religious or spiritual beliefs might be that we asked open-ended questions, whereas most studies that have found these opinions among Vietnamese have explicitly used questions regarding cultural beliefs (43, 48), thus facilitating the expression of cultural beliefs. Other studies were done with people seeking traditional heeling, with traditional healers or religious leaders (45, 49). Furthermore, we didn’t label the protagonist as mentally ill, so participants reacted to the symptoms not to the label. Finally, social desirability might have influenced the answers towards the expression of concepts more in line with Western psychiatry.

One of the rare examples mentioned both supernatural and psychiatric descriptions of the presented case of schizophrenia:

*“Very severe condition. He has a problem with delusion, but might also be possessed or bewitched. He should go to the doctor, or to an exorcist, if not, then he should go to a psychiatry for a few years.” (tình trạng rất nặng, có vấn đề về bệnh hoang tưởng, nhưng cũng có thể bị ma nhập hoặc bỏ bùa. Nên cho đi khám bác sĩ, cho đi trừ tà. Nếu không được thì vào trại tâm thần vài năm).*

Mentioning “madness” *(điên loạn, phát điên)* was rare in our sample, whereas in a study by van der Ham et al. (38) 31% of the participants spoke of “madness”. It is relevant to mention that the samples of these authors were from other regions of Vietnam, Ho Chi Minh City, and Huế and that there might be regional differences.

### Treatment recommendations

We did not ask specifically for treatment recommendations when presenting the vignettes to avoid framing the presented cases as treatable mental illnesses. Yet, many participants suggested at least one possible treatment. 17,9% recommended one kind of medical or psychological treatment. Other types of answers, like the support of surrounding people (n=36), rest (n=11), leisure (n=2), and traveling (n=1), were not often represented.

Most recommended a hospital examination or consulting a medical doctor, while psychological treatment was less recommended. Nevertheless, it is noteworthy that 4,8% (n=51) of all participants suggest those affected should see a psychologist - more often than to see a psychiatrist (0,9%; n=10) or receive medication (n=2). Still, psychological treatment was explicitly named the most regarding a specific treatment for mental illnesses. Participants recommended psychotherapy more often in the depression condition, but a psychiatrist more often in the schizophrenia condition. This suggests the awareness of the difference between the types of practitioners: the terms were not used synonymously. It could be interpreted as a reflection of the fact that psychotherapy has longtime been seen as more helpful for depression than for schizophrenia, which is generally believed to have a stronger biological basis (50–52).

In a questionnaire-based study by Böge et al., seeing a psychiatrist or psychologist were both recommended for schizophrenia by more than 80% of the participants. A higher number of participants recommended psychotherapy than seeing a psychiatrist. Although the percentage of participants recommending this was far lower in our study, which was based on open-ended questions, it is interesting that psychotherapy was frequently mentioned despite the lack of psychologists or psychotherapeutic training in Vietnam. According to a survey by the WHO (6), there were only 0.09 psychologists and 9.1 psychiatrists per 100.000 inhabitants; according to a study by Cuong (53), there were only 120 clinical psychologists at that time in Vietnam. Since then, the number of psychologists has risen, as a master’s program in Clinical Psychology was established in 2009, and ongoing projects to improve psychotherapeutic care have been implemented (54).

Our data appears to reflect a demand for psychotherapy despite the relative absence of large-scale structured psychotherapy programs at the time in Vietnam. This could reflect a growing awareness of the existence of psychotherapy as a treatment method, as a consequence of the ongoing transformation of the mental healthcare system in Vietnam, but most likely is also, more generally, due to globalization and the availability of Western media, social media and streaming platforms etc.

### Gender effects

The male vignettes were more often interpreted as showing signs of psychiatric or psychological disorders, while the state of the female character was more often attributed to stress. Also, the male vignette was more often considered “not normal” than the female vignette. This suggests that the case was perceived to be more severe when presented in a male-gendered vignette. A possible explanation is a common expectation that men must be strong and provide major financial support for their families (55, 56). Tran et al. (57) pointed out that in Vietnamese culture, men should not show negative emotions, and women are less stigmatized for issues with depression. Several other studies have found less mental illness-associated stigma in women (28, 52, 58–60). Mental illness is more likely to be seen as a sign of weakness in men. The fact that in both vignettes, the depicted person’s ability to work was limited might have appeared as a more severe problem in the male condition than in the female condition. However, women’s participation in the workforce in Vietnam is relatively high, at 68,5% (61). Knodel et al. (62) suggests that gender division within the household has shown little change over the last 50 years: gender roles and perceptions are more likely to be continued than changed. According to Hoang (63) women are still seen primarily as mothers and caregivers while labor-force participation has increased, which overburdens women with excessive duties. There might be a “double burden” to shoulder the role of paid labor and the unpaid caregiver, as Dung et al. suggest (64). With this in mind, one could speculate that it is indeed more “normal” to find the described symptoms in the depression vignette more often among women, as our participants expressed.

There was no significant difference in the total amount of recommendations between the male and female vignettes. Still, psychotherapy was recommended significantly more often for the female protagonist, whereas treatment in a hospital or medical center was recommended more often for the male protagonist.

These patterns could be interpreted as a greater concern for mentally ill men but most likely also reflect a higher social stigma attached to mental illness, and thus a barrier to help-seeking behavior in men. Other studies have shown that men are generally more hesitant to seek mental health care in East Asia (65, 66) and have a more negative attitude towards psychiatrists among Vietnamese men (28). Further research is necessary to investigate if the gender differences we found are replicable and, if so, how to address them to reduce potential barriers to help-seeking behavior.

### Limitations

A possible limitation of our study is that participants knew the relation of our research to a research group based at the psychiatric department of Hospital 103, Military Academy of Medicine. This could have led to a social desirability answer pattern so that non-Western, non-medical models of mental illness were less elicited. Furthermore, the distribution through hospital staff members could lead to a sample that is particularly familiar with mental illness. We further distributed the questionnaires through outsiders of the hospital as multipliers to reduce this effect as much as possible. (A randomized sample that contacts participants through a phonebook, for example, was not possible due to the legislation of Vietnam). The fact that 801 (75,2%) of the participants said they did not know anyone who works with mentally ill people in any way strongly suggests that the distribution of the questionnaires has been sufficiently far from the direct environment of the hospital staff who initiated the distribution.

A variety of studies have used vignettes that were developed by Schomerus and Angermeyer (26, 67–70). These vignettes are based on the ICD-10 and DSM-IV. We used the same vignettes to ensure comparability, but they also conform to the DSM-5. Even though the vignettes were not labeled (no mental illness, no diagnosis was mentioned), it is still a presentation of symptoms and behaviors that are described in the DSM-IV. Therefore, it is not a narrative free from Western conceptualizations of mental illness. Although many symptoms of what defines schizophrenia do appear in all cultures (71–73), any vignette presenting a case of “mental illness” is a culturally specific perspective on a certain mental state that could also be narrated differently. Depression has even been described as a culture-bound phenomenon (74), which is hard to distinguish from “ordinary unhappiness or misery” when used to describe forms of distress in non-Western societies (75). The distress that leads to the definition of depression by Westerners could be framed and expressed differently in other cultures. As Kleinmann puts it, “Describing how it feels to be aggrieved or melancholic in another society leads directly into an analysis of a radically different way of being a person”(A. 76). China is often quoted as an example, where similar stressors emphasize somatic symptoms (lack of sleep, poor appetite, and headaches) rather than psychological ones (77, 78). It has been pointed out that psychiatric research is in itself a Western cultural practice (79).

How persons from different sociocultural backgrounds perceive psychiatric vignettes based on the DSM-IV or ICD-10 is nonetheless important because these manuals are internationally recognized and serve as a basis for mainly Western psychiatric conceptualization and as a foundation for the ongoing discourse regarding the basic universality of mental illnesses categories. As mentioned, the symptoms of schizophrenia and depression (if not the practice of grouping them and giving that set of symptoms a specific name) appear to be universal. Psychiatric care is already a common practice in many non-Western societies, and people’s reaction to its concepts affects compliance and stigma (as stated above). As Beck (80) noted, many researchers in the field of transcultural mental health would place themselves along a spectrum between a radical universalist position and the view that Western psychology has nothing or not much to offer to people from non-Western societies. Most of our study participants considered both disorders, schizophrenia and depression, to be an illness (81).

## Conclusion

People in the Hanoi area appear to be familiar with Western concepts on mental health, even if the correct diagnosis from a Western psychiatric point of view was rarely attributed to the vignettes, especially in the schizophrenia condition. The fact that depression was mentioned more often than schizophrenia in both conditions might reflect the impact of the large-scale psychiatric programs that focused on depression (3). The Western psychiatric discourse is spreading with the implementation of mental health projects in Vietnam. The study design might have omitted other local ideas on distress and potential treatments. Still, our data reflects familiarity with Western psychiatry and shows that most participants consider the presented cases to be cases of mental illness. Future research could investigate over the following years if the changes in the mental health care system lead to an improvement in mental health literacy in the sense of “mental illness recognition” and how future developments impact the perception of mental illness in Vietnam.

Vignettes with male protagonists were more likely to be seen as showing signs of mental illness and more often considered “not normal”, which might reflect a difference in stigma attached to mental illness and greater concern for mentally ill men. It would be valuable to investigate further differences in illness recognition and help-seeking behavior (77) between the genders and if there are gender-specific barriers to mental health care in Vietnam.

To address the gap in mental health literacy and the gendered attitudes toward mental health identified in this study, mental health awareness activities should focus on culturally tailored approaches, with direct involvement of individuals with lived experiences of mental illness from different genders, to facilitate personal contact and thus reduce stigmatization (82). Other activities could include cultural, contextual, and language adaptations of the recently launched WHO ‘Mosaic Toolkit to End Stigma and Discrimination in Mental Health,’ developed with the Global Mental Health Peer Network and anti-stigma experts to implement best practices and evidence to improve mental health literacy (83). Given the high penetration of smartphones and the internet in Vietnam, mental health awareness activities can further leverage social media and internet platforms, which are widely used in Vietnam, to disseminate both educational and relatable information on mental health and available services. Additionally, integrating mental health and wellbeing education earlier, for instance, in the school curricula or into workplace mental health programs or involving “patients as educators” (84) in the medical curriculum at medical universities or into newly developed graduate courses on clinical psychology (85) can further normalize conversations and reduce mental health-related stigma both on a public and professional level (86).

## Data Availability

The raw data supporting the conclusions of this article will be made available by the authors, without undue reservation.
